# Wave Dispersion Behavior in Quasi-Solid State Concrete Hydration

**DOI:** 10.3390/s23083947

**Published:** 2023-04-13

**Authors:** Yin Chao Wu, Sanggoo Kang, Yeongseok Jeong, Dafnik Saril Kumar David, Suyun Ham

**Affiliations:** 1 Department of Civil Engineering, The University of Texas at Arlington, Arlington, TX 76019, USA; 2 School of Architecture, Kyungpook National University, Daegu 37224, Republic of Korea

**Keywords:** wave dispersion, wave scattering, hydration, analytical solution, sensors, P-wave, surface wave, inhomogenous medium

## Abstract

This paper aims to investigate wave dispersion behavior in the quasi-solid state of concrete to better understand microstructure hydration interactions. The quasi-solid state refers to the consistency of the mixture between the initial liquid–solid stage and the hardened stage, where the concrete has not yet fully solidified but still exhibits viscous behavior. The study seeks to enable a more accurate evaluation of the optimal time for the quasi-liquid product of concrete using both contact and noncontact sensors, as current set time measurement approaches based on group velocity may not provide a comprehensive understanding of the hydration phenomenon. To achieve this goal, the wave dispersion behavior of P-wave and surface wave with transducers and sensors is studied. The dispersion behavior with different concrete mixtures and the phase velocity comparison of dispersion behavior are investigated. The analytical solutions are used to validate the measured data. The laboratory test specimen with w/c = 0.5 was subjected to an impulse in a frequency range of 40 kHz to 150 kHz. The results demonstrate that the P-wave results exhibit well-fitted waveform trends with analytical solutions, showing a maximum phase velocity when the impulse frequency is at 50 kHz. The surface wave phase velocity shows distinct patterns at different scanning times, which is attributed to the effect of the microstructure on the wave dispersion behavior. This investigation delivers profound knowledge of hydration and quality control in the quasi-solid state of concrete with wave dispersion behavior, providing a new approach for determining the optimal time of the quasi-liquid product. The criteria and methods developed in this paper can be applied to optimal timing for additive manufacturing of concrete material for 3D printers by utilizing sensors.

## 1. Introduction

Concrete is the most widely used construction material due to its high durability and strength with relatively low material cost. It is a heterogeneous material mainly composed of cement, water, and aggregates, and those components are mostly mixed on the construction site. The quality of the hardened concrete depends on the processes of mixing, casting, and curing of the fresh concrete that refers to the early-age concrete before setting occurs [1]. From the quality control standpoint, concrete curing is a significant process for achieving the full potential of the concrete mixture by aiding cement hydration, defined as the chemical reactions between cement components and water contributing to the setting and hardening of fresh concrete [2]. In construction, minimized curing time with a desirable concrete strength to prevent structural failure can reduce the construction time by removing the formwork early. Therefore, understanding and monitoring the hydration process can be the key point in terms of the proper curing time and concrete quality control of 3D printing products. 

“Hydration behavior” refers to the interactions between water and cement particles that affect a material’s properties. During hydration, the material undergoes chemical reactions that alter its physical and mechanical characteristics. The most notable change is the transformation from a liquid or semi-solid state to a durable solid state due to the formation of chemical bonds between molecules [3]. Hydration can also impact properties such as strength, porosity, and permeability. Several factors influence hydration behavior, including moisture content, temperature, chemical composition, and curing time [4]. Moisture content affects the pace and extent of hydration, and inadequate or excessive moisture can lead to erratic or weak structures. Temperature is also crucial, as higher temperatures accelerate hydration while lower temperatures slow it down due to hydration being an exothermic process [5]. The unique chemical composition of materials such as Portland cement adds complexity that experimental and computational methods struggle fully to address [6]. Moreover, curing time requirements vary depending on specific material parameters, and over-curing or under-curing can affect the material’s physical properties and overall quality [7]. 

Recently, hydration has been extensively studied across various fields, including chemistry, biology, physics, and materials science. Studies of the hydration monitoring of cementitious material have gained much attention in the nondestructive testing (NDT) area by using heat-based monitoring [8], ultrasonic pulse velocity test (e.g., with piezoelectric sensor [2], contact ultrasonic transducer [9], noncontact ultrasonic transducer [10]), wave reflection, and wave spectrum analysis [11]. These techniques use the fresh concrete property in terms of the heat and density change, which indicates as the wave velocity change during the hydration process. 

Advancements in 3D printing technology has sparked interest among engineers in the field of 3D printing materials. One area of interest is the hydration process during the drying of cellulose nanocrystals (CNCs), a 3D printing material, which has been studied using neutron radiography [12]. The author found that drying the 3D-printed samples resulted in a decrease in hydration degree and an increase in porosity. Other studies have focused on the microstructure and pore structure during the hydration process, such as the work of Che Y and Yang H, which used X-ray diffraction (XRD), scanning electron microscopy (SEM), and backscattered electron image (BSE) analysis [13]. In 3D printing concrete, a significant challenge is achieving strong inter-layer adhesion, particularly in extrusion-based printing, where flaws can create stress concentrations [14]. Another challenge is developing a thixotropic material that can be easily extruded and resist deformation from subsequent layers [15]. It is essential to thoroughly investigate one of the key factors that can affect the hydration behavior of materials, making further study of this topic valuable. However, despite numerous studies of hydration monitoring, evaluating the hydration process in situ and in real-time remains a challenge using current NDT due to complex microstructures and various factors, including moisture content, temperature, chemical composition, and curing time [16].

The dispersion behavior of fresh concrete during the early stages of hydration is of great interest to researchers. Previous studies have found that strong dispersion occurs as the phase velocity values drop, especially at higher frequencies [17]. This suggests that the microstructural effects responsible for the dispersion of fresh concrete when in liquid form are reversed after the transformation of the medium into a hardened solid state. However, it remains to be seen whether this theory can be applied for real-time monitoring as the material evolves over time. 

This paper proposes suggestions to overcome key technical barriers for understanding comprehensively wave dispersion in quasi-solid states during early hydration processes with three main objectives. Firstly, we measured the wave scattering behavior of P-waves and surface waves using both contacted transducers and noncontact sensors. Secondly, we studied the dispersion behavior in fresh concrete samples with various impulse frequencies. Finally, we compared laboratory data with analytical solutions to better understand wave dispersion behavior in quasi-solid states. During the initial stages of hydration, changes in the microstructure of the quasi-liquid product in fresh concrete may occur over time. Therefore, the objective of this study is to comprehensively understand the dispersion behavior of fresh concrete during the hydration process, including the changes in microstructure that occur during this time. This study provides important insights into the development and performance of cement-based materials.

## 2. Theory 

**Figure 1:**
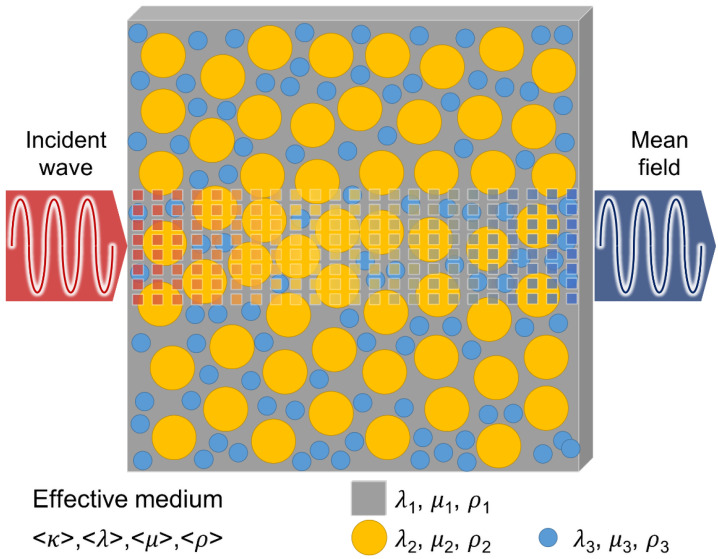
Schematic of wave propagation in an inhomogeneous effective medium.

Several researchers have observed that at higher frequencies, there is a decrease in phase velocity values, resulting in significant dispersion. This phenomenon has been noted in previous studies [17]. From the study, the microstructural effects that cause dispersion in fresh concrete when it is in a liquid state are reversed after the medium has transformed into a hardened solid state. During early hydration, there are possible changes in microstructure in the quasi-liquid product in fresh concrete over time. This study focuses on understanding dispersion behavior during the hydration process. Thus, this section briefly describes the formulation and solution of the single-scattering problem. The present approach is based on the Ying and Truell (1956) formulation considering scattering of a plane wave on an elastic sphere embedded in an infinite elastic matrix [18]. The ultrasonic mean field propagating and scattering behavior can be explained by the effective medium scheme [19]. There are three different phases in mortar including solid phase (cement matrix), elastic inclusions (aggregate), and air voids, as shown in Figure 1.

The dispersion behavior of mortar is assumed if the aggregate size is much larger than the characteristic capillary pore size but smaller than or comparable to the ultrasonic wavelength [20]. The effective longitudinal complex wave number k(ω), which is calculated from the longitudinal phase velocity $V_{L}(\omega )$ and the effective longitudinal attenuation $\alpha_{L}(\omega )$, can be used to describe the wave propagation through the composite material:

(1) $$k\left(\omega \right)=\frac{\omega }{V_{L}(\omega )}+i\alpha_{L}(\omega )$$

The Equation (1) can be expressed by the effective elastic modulus constants such as Lamé modulus (λ), shear modulus (μ), and the effective density (ρ):

(2) $$k\left(\omega \right)=\omega \sqrt{\frac{\rho }{\lambda +2\mu }}$$

Several indicators point to lossless redirective scattering as the dominant mechanism in wave propagation within the material being considered [20,21]. When waves encounter an obstacle or interface between different media, they can be redirected or scattered in various directions. This phenomenon is known as wave scattering, and it occurs because waves interact with the surface of the object they encounter [18,22]. The simple multiple scattering theory is proposed to describe the phenomenon when a pulse propagates through a particulate composite material, interacting with the embedded particles and undergoing both dispersion and attenuation [20,23].

Scattering on a spherical obstacle has been extensively discussed in the literature, so this discussion will provide only general guidelines [23,24,25,26,27,28]. When a compressional wave encounters a particle, it generates both compressional and shear waves inside the particle, as well as scattered compressional and shear waves outside the particle. The wave dispersion model by the multiple scattering model is considered an effective medium, and the size of inclusions distribution can be expressed by [18,19,22,23,28]:

(3) $${\left(\frac{K(\omega )}{k_{c1}}\right)}^{2}=1+\frac{3\varphi }{{k_{c1}}^{2}R^{3}}f\left(0\right)+\frac{9\varphi^{2}}{{{4k}_{c1}}^{4}R^{6}}[f^{2}\left(0\right)-f^{2}\left(\pi \right)]$$

where $k_{c1}$ is longitudinal wave number of the matrix and φ and R are the particle volume fraction and its radius, respectively. $f\left(0\right)$ and $f\left(\pi \right)$ are the complex forward and backward amplitude, respectively. When modeling the scattering of particles suspended in a liquid, the equations can be derived using a limiting process in which the shear modulus of the host medium approaches zero [23,29]. In the present formulation, temperature and heat transfer effects are not included.

The $f\left(0\right)$ and $f\left(\pi \right)$ can be expressed by [29]:

(4) $$f\left(0\right)=\frac{1}{{ik}_{c1}}\sum_{n=0}^{\infty }\left(2n+1\right)A_{n}$$

(5) $$f(\pi )=\frac{1}{{ik}_{c1}}\sum_{n=0}^{\infty }{\left(-1\right)}^{n}(2n+1)A_{n}$$

where $A_{n}$ is one of the scattering coefficients based on different boundary conditions and the Henkel and Bessel function [18,22].

## 3. Method

To comprehensively study the wave dispersion phenomenon in quasi-solid states during early hydration processes, this study has three main objectives. Firstly, we measured the wave scattering behavior of P-waves and surface waves using both contacted transducers and noncontact sensors during the early hydration process. We calculated the phase velocity of P-waves and surface waves using the dispersion behavior caused by random scattering in a quasi-solid-state medium (as shown in Figure 1). We used two different wave distance experimental setups at various frequencies ranging from 40 kHz to 150 kHz. This study aimed to obtain the hydration relationship in quasi-solid states. Secondly, we studied the dispersion behavior in experiments using fresh concrete samples prepared with a mixture of cement, sand, and aggregate. We examined a fixed proportion of aggregate with different water-to-cement ratios (w/c) and different proportions of aggregate with w/c = 0.5. Thirdly, we compared the phase velocity of the dispersion behavior calculated from laboratory data with analytical solutions. Analytical solutions provide cleaner results without environmental factors. The difference between the analytical solutions and the laboratory results was analyzed to better understand the wave dispersion behavior during the early hydration process in quasi-solid states. This approach allows for a detailed understanding of the wave dispersion phenomenon during the early hydration process, providing insights into the development and performance of cement-based materials.

First, to understand wave dispersion in quasi-solid states, we continuously recorded experimental data every five minutes from 5 to 30 min after mixing. Two types of wave dispersion testing were performed: P-wave testing using contact transducers and surface wave testing using noncontact sensors. In general, wave dispersion behavior is investigated with the phase velocity when a pulse travels through the medium. The phase velocity is a critical indicator of quasi-solid states and can be used to monitor changes in materials during the hydration process, particularly the transition from a liquid to a solid state.

**Figure 2:**
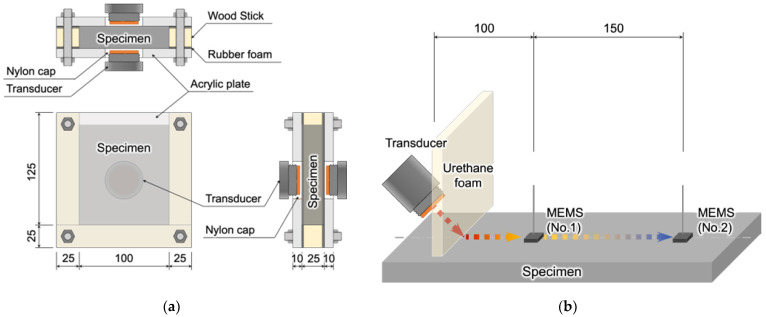
Setup for testing (**a**) contact P-wave and (**b**) noncontact surface wave.

To determine the phase velocity of P-waves, the different wave path distance of the specimen needed to be measured using different testing configurations. For P-wave testing, two wave path designs of 1-inch (2.54 cm) and 2-inch (5.08 cm) were utilized, as shown in Figure 2a. The contact transducers used in this study were V601 from Olympus. The container was constructed with two parallel side walls made of transparent polymethyl methacrylate. In the center of the wall, a circular insert nylon wear cap was used to protect the transducer from direct contact with the mixture. The surface wave test utilized a noncontact sending transducer (NCG50-D50, Ultran) and noncontact receiving sensors consisting of multiple micro-electromechanical systems (MEMS) (SPU0410LR5H, Knowles), as illustrated in Figure 2b. To accurately record the surface wave dispersion, the distance between the two MEMS was set to 10 cm. To more easily describe the P-wave and surface wave results, S1 and S2 denotes the P-wave 1-inch and 2-inch thickness specimen and MEMS denotes the surface wave results.

The P-wave test calculates the phase velocity by measuring the time delay caused by the wave dispersion of the target wave between two specimens with different wave propagation distances. Meanwhile, the surface wave test calculates the wave delay by the distance of each MEMS sensor location.

The phase velocity of a P-wave and a surface wave is calculated from the time delay of the propagated wave between the two specimens (Δ*t*) and the distance of the sensor (Δ*d*, 1-inch difference thickness in P-wave, and 1-cm in surface wave). The expressions can be displayed as:

(6) $$V_{phase}\left(\frac{m}{s}\right)=\frac{\Delta d(m)}{\Delta t(s)}$$

**Figure 3:**
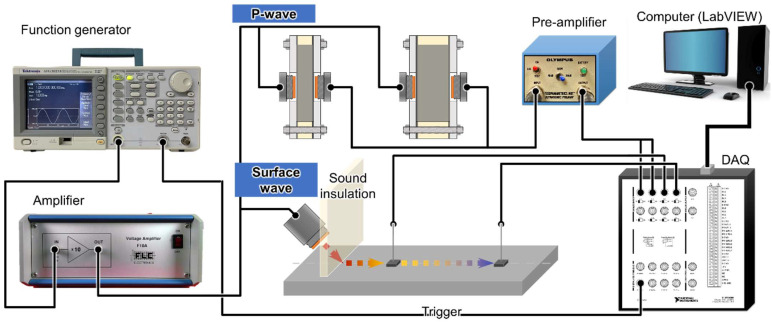
Overall testing configuration of both P-wave and surface wave.

Figure 3 illustrates the overall equipment and testing configuration used in the experiment. The wave pulse was generated using the AFG3021B function generator manufactured by Tektronix, which enabled pulse transmission within the frequency range of 25 MHz. The pulse waveform consisted of a three-cycle wave, and the sending interval was fixed at 5 milliseconds to prevent continuous pulse transmission into the medium, which could potentially disrupt the dispersion waves. A high voltage amplifier (F10A, Acquitek) was utilized to increase the pulse amplitude from the function generator. The data acquisition system was produced by National Instrument (USB-6366, NI). Additionally, another pre-amplifier (5676, Olympus) was used to increase the amplitude of the received signal, as the wave’s amplitude may attenuate while passing through the medium.

## 4. Results

This section presents two main results: firstly, an analysis of the raw data under various scanning times with different pulse frequencies, and secondly, an investigation of the phase velocity of P-waves through the use of analytical solutions and experimental data. The analytical solution assumes wave dispersion with particles in a mature medium, while the experimental data focuses on the early-age medium to determine the effect of microstructure in the quasi-solid state. By comparing the analytical solution with the experimental results, the dispersion behavior within the early age of the medium can be determined.

### 4.1. Exploring the Behavior of Wave Dispersion

**Figure 4:**
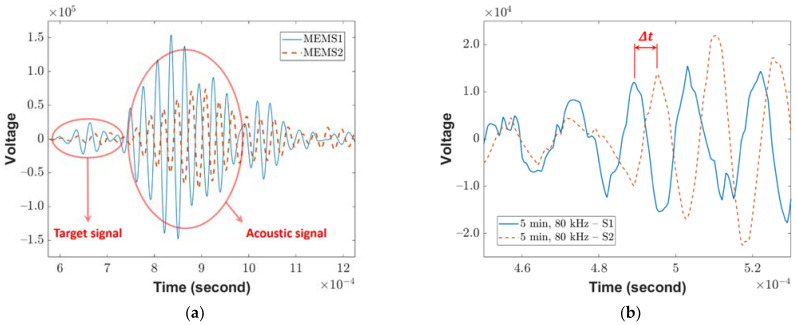
Raw data obtained from (**a**) surface wave experiment with 80 kHz impulse, five minutes after mixing concrete and (**b**) the raw data of P-wave S1 and S2. The ∆t is calculated from the first peak of each sensor.

Figure 4a displays the raw surface testing data obtained using an 80 kHz impulse, five minutes after mixing the concrete. The waveforms exhibit two distinct groups: the first group represents the target wave, and the final group corresponds to the reflected signal. As the impulse interval is five milliseconds, which is longer than the two-millisecond recording time, it assists in determining the arrival time of the target signal. To calculate the phase velocity, the target signal is analyzed without considering the reflected area. This is because the previous pulse wave reflection may have already distorted the wave dispersion. The time delay ∆t of first peak of P-wave as shown in Figure 4b.

**Figure 5:**
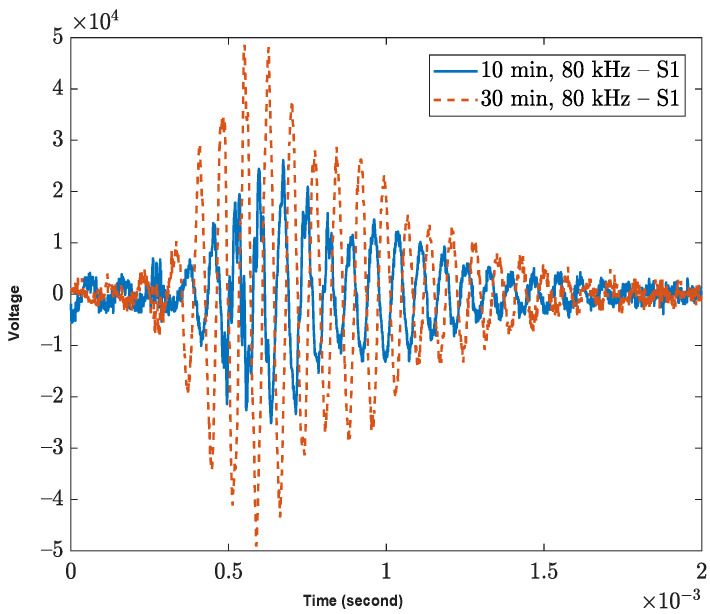
Comparison of wave speeds in concrete specimen during hardening process using different scanning times. The wave speed in the 30-min scanning data is faster than the 10-min data due to the ongoing hydration process of the concrete.

The phase velocity is determined using Equation (6), with a Δd of 1 inch (2.54 cm) for the P-wave setup and a Δd of 6 inches (15.24 cm) for the surface wave. Additionally, the data obtained at different scanning times can reveal the wave dispersion or wave stretching due to multiple scattering in the medium. Figure 5 shows the data collected 10 and 30 min after mixing the concrete specimen. The wave speed is faster in the 30-min data, as the concrete has started to harden in comparison to the 10-min data. The stretching effect implies that the phase velocity may increase as the specimen undergoes the transition from a liquid to a solid state.

### 4.2. Analysis of Phase Velocity Variation Due to Dispersion

**Figure 6:**
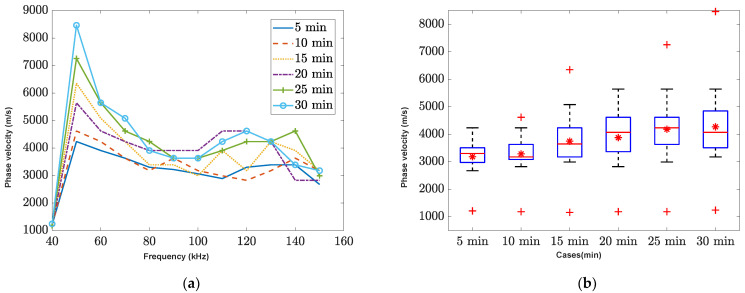

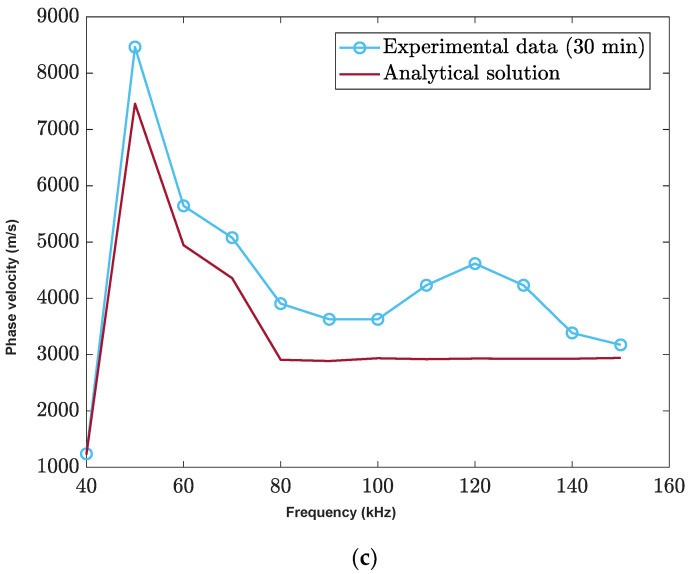
The phase velocity of P-wave experimental data analysis. (**a**) phase velocity with six different scanning time, (**b**) the boxplot of different time cases, and (**c**) the comparison between analytical solution and experimental data.

The changing phase velocity can be used to represent the dispersion behavior of the mortar specimen during the hydration process, which involves a transition from a liquid to a solid state. Figure 6a,b shows the pairs of experimentally obtained phase velocity and frequency for fresh concrete over time, with six different hydration stages exhibiting a similar trend. Strong dispersion is observed as the phase velocity values decrease at higher frequencies. The maximum phase velocity occurs at 50 kHz for all cases and gradually decreases as the impulse frequency increases. At lower frequencies of incident wave, longer wavelengths of the impulse wave can pass through the particle without significant reflection or disturbance. However, at higher frequencies, random reflected waves can interfere and cause more disturbance.

Among the six cases studied, the 5-min data exhibited the lowest wave speed, indicating stronger wave dispersion irrespective of incident frequency. It can be generally concluded that stronger dispersion is observed for less stiff material stages, such as those with a high-water content, and also during the earliest stage of hydration, which has a high impedance mismatch (e.g., aggregate vs water-cement paste). This may be attributed to the presence of more liquid in the mixture during the early stages, which can affect the speed. The box plot presents statistical values (minimum, maximum, average, and median) for each scanning time data. Overall, the average and maximum phase velocity increased over time, suggesting that dispersion behavior mostly occurs during the early stages of the mixture.

The comparison between the analytical simulation and the experimental data is shown in Figure 6c. The analytical model did not consider any interference or random reflected waves, resulting in a flat phase velocity at higher frequencies. The maximum speed was observed at 50 kHz and decreased at higher frequencies. However, the current analytical solutions present a challenge as they do not include a time input function in the equation, making it difficult to explore the system’s behavior over time. To address this limitation and investigate the system using different time analytical solutions, our future plans include the development of analytical models that incorporate a time function.

**Figure 7:**
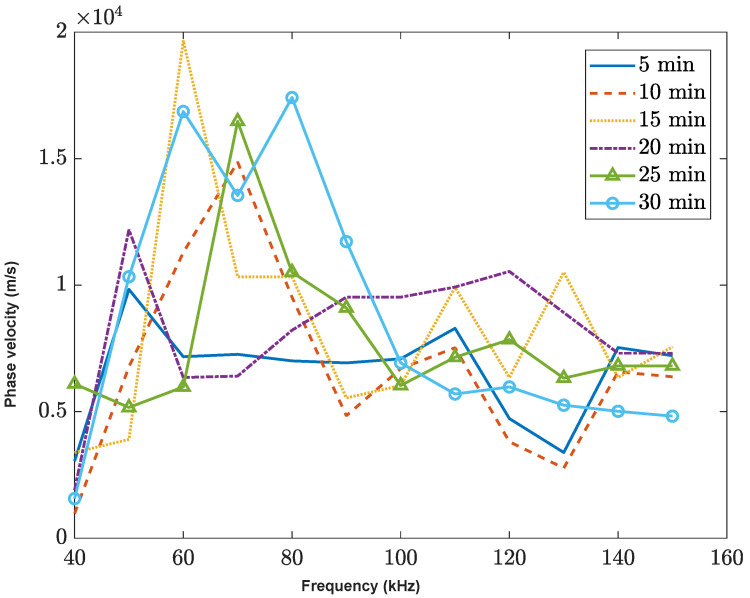
The phase velocity of surface wave experimental data analysis. The phase velocity with six different scanning times.

The phase velocity of the surface wave as shown in Figure 7. Similar with P-wave experimental data, the phase velocity of the surface wave has a higher value in the low-frequency impulse range within 50 kHz to 80 kHz, while the phase velocity decreases in the high-frequency range. Additionally, the later stage of the hydration at 30 min exhibits a higher average velocity across all frequency cases. Comparing the contacted sensor data (P-wave) with the non-contacted sensor data (surface wave) of the dispersion behavior, both have similar trends of phase velocity peak with a certain frequency range. There is a need for further study of dispersion of surface waves and leaky waves obtained from noncontact sensors. Moreover, there is currently no analytical solution that helps to explain the dispersion behavior of surface waves. As a result, this paper presents experimental data that demonstrate the dispersion pattern over time. Further studies and comparisons of surface waves will be included in our future plans.

## 5. Conclusions

This paper investigates wave dispersion behaviors using both contact P-wave and noncontact surface wave techniques. The phase velocity of the P-wave experimental results is compared with the wave dispersion analytical solution contributed by other researchers. Both the P-wave experiment and the analytical solution exhibit a maximum peak with a 50 kHz impulse, while the experiment is more affected by microstructure dispersion with a higher frequency impulse. The phase velocity at 5 min is the lowest because the medium is still inhomogeneous and close to a liquid state, which causes most of the wave energy to be attenuated and results in a lower wave speed. The surface wave exhibits more varied patterns over time. However, an analytical solution for surface wave dispersion has not yet been developed, so a conclusion regarding the surface wave cannot be made at this time. Developing an analytical solution for surface wave dispersion will be a task for future research. Based on our findings, the following conclusions can be drawn:

- As concrete changes from a fresh, liquid state to a hardened state, its microstructure changes, indicating that wave dispersion behavior is affected by the hydration process.
- In the contact sensor P-wave study, both the analytical solution and the experimental data indicate a similar trend, with higher phase velocities observed at 50 kHz and lower speed in higher frequencies.
- In the noncontact sensor surface wave study, a faster speed is observed in the lower frequency range.
- The observed increase in phase velocity over time suggests that the hardening progress of hydration directly affects wave dispersion.
- Since the current knowledge of analytical solutions is limited, future studies should focus on developing a more comprehensive analytical solution for P-wave dispersion over time. This will enable the demonstration and explanation of an ideal early-age dispersion P-wave without any reflected situations.
- Future experiments will include investigating the effects of temperature and the different aggregate proportions on the results.

## Data Availability

Not applicable.
